# The growing and urgent need for interventions to mitigate cancer-related stigma in sub-Saharan Africa

**DOI:** 10.1017/gmh.2026.10252

**Published:** 2026-07-20

**Authors:** Huda Haque, Brandon Knettel, Judith Mwobobia, Megan J. Huchko, Kim Madundo, Furaha Serventi, Blandina Theophil Mmbaga, Kristin Schroeder, Nosayaba Osazuwa-Peters

**Affiliations:** 1 Duke Global Health Institute, Duke University, USA; 2 Duke University School of Nursing, USA; 3 The Brown School, Washington University in St Louis, USA; 4 Duke University, USA; 5 Department of Psychiatry, Kilimanjaro Christian Medical Centre, United Republic of Tanzania; 6 Department of Oncology, Kilimanjaro Christian Medical Centre, United Republic of Tanzania; 7 Kilimanjaro Christian Medical Centre, United Republic of Tanzania; 8 KCMC University, United Republic of Tanzania; 9 Kilimanjaro Clinical Research Institute, United Republic of Tanzania; 10 Department of Oncology, Bugando Medical Centre, United Republic of Tanzania; 11 Duke University Division of Otolaryngology Head and Neck Surgery, USA; 12 Duke University Department of Population Health Science, USA; 13 Duke Cancer Institute, USA

**Keywords:** cancer, stigma, mental health

## Abstract

Cancer stigma, both underaddressed and understudied, remains a pervasive challenge in sub-Saharan Africa. Currently, the region is experiencing rising cancer incidence alongside some of the lowest cancer survival rates worldwide. With complex and multifaceted sociocultural factors contributing to this stigma and subsequent lack of screening, low treatment adherence and poorer health outcomes, it is critical to implement effective interventions in the region, at both the clinical and policy levels. To achieve a path forward, it is necessary to create targeted efforts that incorporate existing infrastructure and key cultural contexts.

## Graphical abstract

**Figure:**
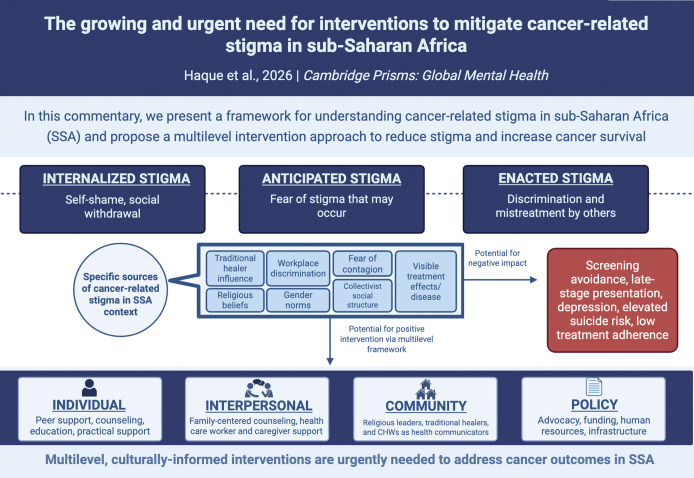

## Impact statement

Cancer stigma is a hidden but consequential barrier to the diagnosis and treatment of cancer in sub-Saharan Africa, driven in no small part by its significant effects on mental health. With the incidence of cancer rising, interventions that address the multifaceted drivers of stigma are urgently needed. Despite the timely nature of this issue, there remain significant deficits in stigma-focused care across the region. This article highlights that stigma operates alongside myriad cultural and social structural factors that must be addressed in culturally sensitive ways to be effective. By synthesizing evidence across cultural, psychological and structural aspects of stigma, this article provides a comprehensive examination of a driving force behind increased cancer mortality. These insights can help guide policymakers, clinicians and global health professionals in designing multilevel interventions and supporting the lives of patients with cancer in sub-Saharan Africa.

## Introduction

Cancer-related stigma is increasingly recognized as a significant yet largely unaddressed influence on cancer mortality due to stigma-driven screening avoidance, late presentation, low treatment adherence and worsened quality of life (Corrigan et al. 2020a). Specifically, people affected by cancer in sub-Saharan Africa face a complex and interrelated set of challenges in a unique sociocultural environment, specifically in low-resource settings marked by inadequate infrastructure and lack of availability of high-quality cancer care (Tessema et al., 2022). While service-delivery factors are undoubtedly major contributors to poor outcomes, challenges are further exacerbated by pervasive stigma (Bray et al., 2024). Notably, cancer incidence is rapidly rising, and survival rates in sub-Saharan Africa are among the lowest globally (Henry et al., 2023). Addressing cancer stigma is therefore consistent with global mental health policy priorities, including the WHO’s Comprehensive Mental Health Action Plan 2013–2030, which calls for the integration of mental health into broader health systems and the reduction of stigma as a barrier to care-seeking (World Health Organization, 2021). In sub-Saharan Africa, stigma-reduction interventions that leverage existing community infrastructure represent a scalable pathway for expanding mental health support within cancer care systems.

Stigma can operate in tandem with structural limitations, intensifying delays in recognition and treatment-seeking behavior. About 85% of cancer patients report experiencing at least one type of stigma (Squiers et al., 2021), and stigma can worsen psychological outcomes in cancer patients (Yilmaz et al., 2019). Specifically, in one study, negative attitudes toward cancer were associated with an ~2.5-fold higher likelihood of depression (Cho et al., 2013). Ultimately, worsening mental health and delayed diagnoses associated with stigma may contribute to excess mortality among cancer patients (Chang and Lai, 2022), underscoring the urgency of addressing cancer stigma in the sub-Saharan African region.

In this context, the integration of stigma-targeting care faces unique challenges, but these can be addressed through a multilevel, culturally competent framework. Doing so is essential for improving survival rates and mental health, and for promoting health equity among cancer patients on a global level. This perspective synthesizes existing literature on cancer stigma in sub-Saharan Africa to inform multilevel interventions. Multilevel intervention frameworks recognize how issues operate simultaneously at different levels and how effective mitigation strategies require tailored approaches at each level. Rather than employing a formal systematic search, this perspective draws on relevant literature to identify actionable priorities to address cancer stigma in the region. The proposed framework draws on the Social-Ecological Model (SEM), which conceptualizes health behaviors as shaped by interacting individual, interpersonal, community and policy-level factors (Bronfenbrenner, 1979; McLeroy et al., 1988). This model has been successfully applied to stigma-reduction efforts in HIV and other chronic illness contexts in sub-Saharan Africa (Henry et al., 2023) and is well-suited to address the multilevel drivers of cancer stigma described in this perspective.

## Cultural and societal drivers of stigma in sub-Saharan Africa

**Figure 1.**
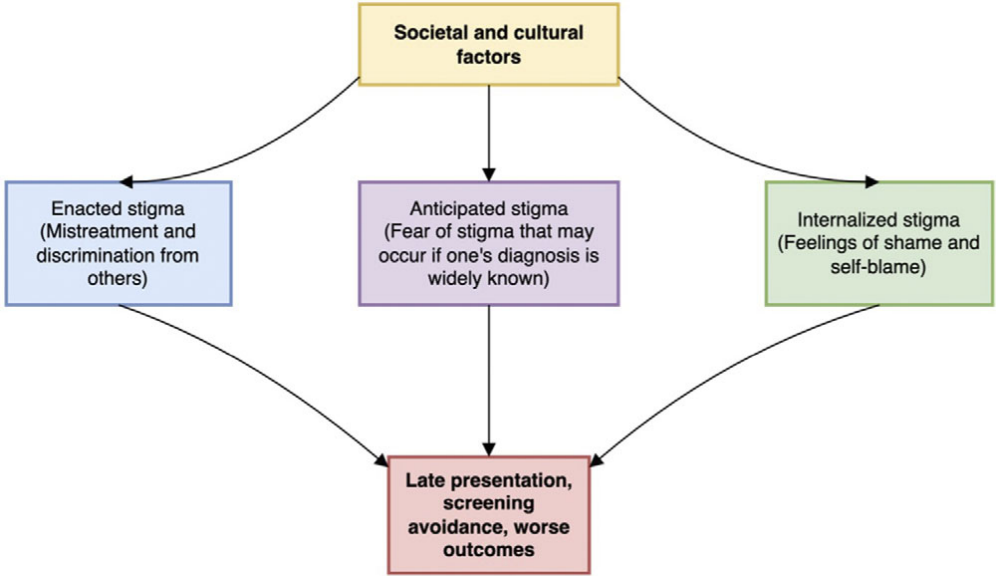
Conceptual model demonstrating how societal and cultural factors can cause three types of cancer-related stigma and subsequent worse health outcomes. Figure 1. long description.

Stigma is defined as a social process characterized by labeling, negative stereotyping and power asymmetry (Andersen et al., 2022). Stigma is a multilevel challenge and can be conceptualized as *internalized*, or self-stigma, *enacted*, involving actual mistreatment and discrimination by others and *anticipated*, involving the fear of stigma that might occur if one’s diagnosis is shared (Knettel et al., 2024; Mwobobia et al., 2024) (Figure 1). Internalized stigma often involves cancer patients experiencing feelings of shame, withdrawing from social life due to their cancer, feeling uncomfortable sharing their diagnosis or actively hiding their diagnosis (Squiers et al., 2021). Enacted stigma can affect patients through discrimination in employment, housing or even physical harassment. Anticipated stigma places a significant mental burden on cancer patients due to their anxieties about being devalued by their social network (Earnshaw et al., 2012). Furthermore, anticipated stigma and the associated strain on a cancer patient’s mental health can dissuade open communication with healthcare providers, thus delaying effective treatment (Chapple et al., 2004). These different dimensions of stigma interact to create a psychological burden that is often cyclical and far-reaching, shaping mental well-being, quality of life and cancer outcomes, including depression, social withdrawal, workplace discrimination and treatment dropout (Yilmaz et al., 2019; Amini-Tehrani et al., 2021).

In the context of sub-Saharan Africa, stigma around cancer is often driven by deeply rooted cultural misconceptions about the illness – for example, there are prevalent beliefs that religious punishment manifests as cancer, or that the disease can be contagious, spread through food or clothing (Maingi and Arch, 2025). In fact, one study showed that cancer is sometimes viewed in sub-Saharan Africa as the consequence of being “bewitched” (Oystacher et al., 2018). Ultimately, these falsehoods may be associated with increased social isolation and mental stress in cancer patients. These notions may be reinforced by inaccurate messaging from traditional or religious healers within sub-Saharan African communities (Mwobobia et al., 2024). Unlike the formal healthcare system, these healers are unregulated, yet they are key leaders who hold considerable influence and often serve as the first point of contact for medical advice. Notably, more people seek traditional medicine to treat cancer in Africa than in any other global region (James et al., 2022), making thoughtful engagement with healers essential.

Cancer stigma and associated mental distress are particularly common in cancers associated with sexual and reproductive health, such as cervical cancer, penile cancer and anal cancer. These cancers often carry connotations of socially stigmatized behaviors such as promiscuity or homosexuality (Gugliotti and Miskolci, 2024). For example, HPV infections and associated cervical cancer are often seen as the consequence of women’s unfaithfulness, and one significant barrier to screening for anal cancer for men is discomfort surrounding disclosure of sexual experiences (Sam et al., 2025). These associations can lead to intersectional stigma: layered discrimination based not only on health status but also on other aspects such as sexuality and gender norms (Mwobobia et al., 2025). Cancers like breast cancer and testicular cancer may be perceived as threatening the traditional concepts of femininity, masculinity, fertility and virility that may significantly shape one’s sense of self and, thus, their mental state (Dax et al., 2022). These negative perceptions, driven primarily by societal norms, can lead individuals with cancer to conceal symptoms or delay care due to worries about the dissolution of intimate relationships (Zarei et al., 2025). Thus, stigma may influence not only health behaviors but also disrupt family structures, adding to the mental health burden on patients.

Beyond these psychosocial effects, visible symptoms and treatment side effects are also highly stigmatizing, including visible lesions, hair loss and surgical outcomes such as mastectomy. A survey of breast cancer patients in sub-Saharan Africa found that mastectomies are seen as mutilation and as processes that leave a woman “incomplete” (Ogunkorode et al., 2021). These physical changes, which often serve as involuntary disclosures of a person’s cancer status, are likely to increase social isolation, impact intimacy and lead to feared or real abandonment by one’s partner (Mwobobia et al., 2024, 2025). Left unaddressed, these fears can evolve into long-term mental health issues and may delay or prevent treatment altogether.

As culture, rural/urban setting and education vary across sub-Saharan Africa, cancer stigma is unlikely to be uniform across the region. While there exists limited literature on the differences in cancer stigma between countries in sub-Saharan Africa, research shows marked differences in HIV-related stigma incidence between sub-Saharan African countries (Teshale and Tesema, 2022). Interventions must therefore involve tailored input from local health officials and community members.

## Cancer stigma has been unaddressed in sub-Saharan Africa

Unlike HIV, which is endemic in sub-Saharan Africa and has a comparatively well-described stigma literature, there is a noticeable paucity of both observational and interventional data on cancer stigma in the region (Corrigan et al. 2020b). We recently conducted a systematic review of research examining cancer stigma in Africa and identified a total of 98 studies on the topic, including distinct focus areas on adult cancers, pediatric cancers and HPV and cervical cancers (Mwobobia et al., 2025). Disappointingly, only two of these identified studies described a stigma reduction intervention, both of which were brief educational interventions for improving knowledge and reducing stigma related to cervical cancer (Rosser et al., 2015; Nkwonta et al., 2021). Additionally, neither study provided support directly to people with cancer. These findings and the lack of longitudinal interventions represent a significant research gap in a region with an increasing incidence of cancer and a missed opportunity to address a growing burden of disease through effective interventions. This gap additionally reflects a larger pattern of underfunding for psychosocial oncology interventions across the region.

This gap in intervention research also reflects a broader lack of attention toward psychological care for cancer patients. Mental health and oncology are not commonly integrated in a sub-Saharan African context. In fact, one systematic review found that the prevalence of optimal, multidimensional cancer care in sub-Saharan Africa was low, with nearly two-thirds of cancer patients reporting unmet needs (Bore et al., 2024). Expanding this evidence base is essential for guiding sustainable, scalable programming.

Cancer stigma also has direct financial and social consequences. Survivors may suffer high medical expenses, loss of income or loss of employment due to discrimination, leading to feelings of burden and a desire for hastened death (Mwobobia et al., 2024), which can worsen or trigger depression and anxiety. In sub-Saharan Africa, where collectivist ideals prioritize communal harmony and familial viewpoints (Appiah et al., 2024), the loss of social support networks due to stigma is especially damaging. Patients may struggle to pursue treatment that risks alienating them from their community. These anticipated threats to social status may significantly increase the risk of severe mental health consequences such as depression and anxiety, both of which are critical comorbidities with cancer (Alwhaibi et al., 2023). In fact, among the 20 million people who will be newly diagnosed with cancer in 2025 globally, it is estimated that 30,000–40,000 of these individuals will die by suicide (Rafiei et al., 2024). Specifically, suicide mortality in cancer patients is up to four times higher than in the general population, depending on cancer type (Osazuwa-Peters et al., 2018). Remarkably, there is no data estimating the prevalence of suicide among people with cancer on the African continent, a striking gap given the growing incidence of cancer in the region. This gap means that policymakers have little incentive or guidance to pursue evidence-based policy solutions that effectively address the issues people with cancer face in a structural and sustainable manner. Thus, the relative lack of attention to cancer stigma in sub-Saharan Africa poses significant challenges to developing comprehensive, evidence-based interventions.

## Developing a multilevel intervention to address cancer stigma in sub-Saharan Africa

### Individual interventions

**Figure 2.**
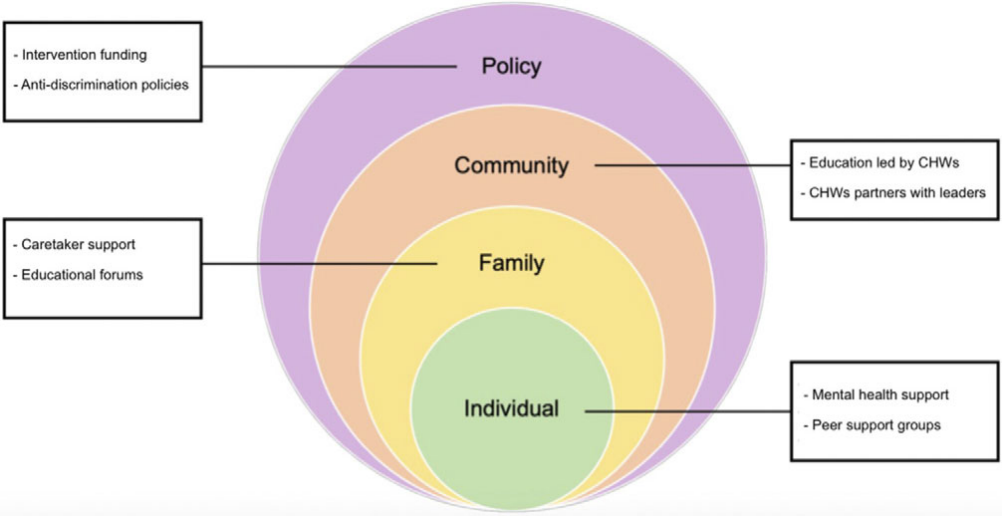
Socio-ecological model of stigma-reduction interventions illustrating multilevel targets for addressing cancer stigma. Figure 2. long description.

Given that the drivers of stigma in sub-Saharan Africa are deeply embedded in cultural and societal norms, effective interventions will likely need to be both multilevel and multifaceted (Figure 2). This includes four categories of intervention that target stigma: individual, interpersonal, community and policy interventions. At the individual level, interventions should focus on reducing internalized stigma through education and mental health support. The awareness of shared experiences can be therapeutic, reducing the feelings of internalized shame and isolation that often accompany a diagnosis. Peer support for individuals with cancer has been shown to improve quality of life outcomes and even extend survival as participants exchange coping strategies and witness examples of recovery (Holtz et al., 2024). Methods such as forums and open dialogues facilitated by mental health professionals or trained social workers have been proven to reduce mental distress (Li et al., 2025). Efforts can foster a sense of individual empowerment for cancer patients to seek treatment and educate their own families, and reduce internalized stigma, with important implications for survivorship.

In contexts such as sub-Saharan Africa, where individual access to psychiatrists is relatively limited, peer networks and interactions can serve as sources of consistent support and individual mental strength (Atewologun et al., 2025). This objective may be achieved through the recital of shared experience and storytelling, which are both effective and tested means of oral communication, knowledge transmission and fostering of collective resilience in many African settings.

### Interpersonal interventions

While addressing the needs of people with cancer themselves is undoubtedly essential, sustainable interventions to combat stigma should incorporate a holistic approach and thus address the family of a cancer patient as well. Research shows that there exists limited psychosocial support for the families of cancer patients in sub-Saharan Africa (Guan et al., 2023). Family-centered stigma-reduction interventions will likely be effective in many sub-Saharan African settings due to prevailing collectivist ideals in various parts of the region. Mental health support for family members, either in a clinic setting or through home visits, can help facilitate stronger social networks for the patient while preventing misinformation or resentment. These interventions can take the form of support groups or counseling sessions led by healthcare workers. Additionally, involving families in interventions can prevent caretaker burnout (Wang et al., 2025). Ultimately, the feasibility of family-centered approaches will vary depending on context, including health systems capacity, the availability of trained facilitators and familial norms.

### Community interventions

Education and cancer awareness interventions may help to mitigate stigma and decrease misconceptions related to cancer. Key to this will be evidence-based, culturally informed community engagement and education of traditional healers and religious leaders, whose established trust with their communities can help them serve as effective public health communicators (Oystacher et al., 2018; Mwobobia et al., 2024; Mwobobia et al., 2025). These community leaders can be trained to become strategic partners to disseminate cancer-related health information and counter disinformation, making substantial inroads with sub-Saharan African communities. These initiatives may contribute to increases in cancer screening, early treatment, improved engagement with care, better mental health among cancer patients and ultimately higher survival rates (Mwobobia et al., 2024). This intervention model has been shown to be successful in the case of HIV-related stigma and could be adopted to address cancer stigma (Henry et al., 2023). Having community leaders explain stigma via traditional and social media can also be helpful and cost-effective, especially for those uncomfortable with the relatively public nature of open forums or without the ability to attend in-person interventions (Metwally et al., 2017).

Additionally, given the absence of a readily available public health workforce and the relative shortage of mental health professionals in many sub-Saharan African settings, task-shifting to include community health workers will be critically important in establishing community-level interventions. Community health workers are vital in low-resource settings, serving as a critical link between the community and formal care systems (Anstey Watkins et al., 2021). In fact, nearly 80% of the sub-Saharan African population relies heavily on community health workers for care (Idriss-Wheeler et al., 2024). They can be particularly effective for populations that perceive formal healthcare settings as stigmatizing (Carrara et al., 2023). Therefore, future interventions should include these valued and trusted members of the healthcare workforce in this region to help address distrust regarding the formal healthcare system. These workers can be equipped not only with clinical skills but also with foundational mental health literacy (Barnett et al., 2018). This training may enable them to recognize signs of mental health distress and dispel any misconceptions that drive community-based stigma and associated issues. Factors such as local health system capacity and the availability of CHWs will be important determinants of the effectiveness of interventions.

### Policy interventions

Finally, policy-based interventions to address discrimination against cancer patients in the workplace, fund community-based interventions and promote widespread public education campaigns throughout the sub-Saharan African region are critical. While community health workers and traditional healers have great potential to reduce stigma in sub-Saharan Africa, structural constraints must be addressed. Many sub-Saharan African health systems face chronic underfunding, attrition of CHWs and a limited capacity to integrate new training/programming. Therefore, dedicated and sustained financing is needed to ensure the effectiveness of stigma-reduction interventions and retention of CHWs. Policymakers can help integrate stigma-reduction training into existing CHW programs to strengthen trust while simultaneously remaining cost-effective (Miyares et al., 2026). Additionally, strengthening anti-discrimination protections in employment can help mitigate enacted stigma (Stergiou-Kita et al., 2016). Such protections can simultaneously reduce stigma on a societal level while ensuring patients are well-equipped to manage financial toxicity associated with treatment and the potential psychological stress of feeling burdensome. Framing stigma reduction as a cost-effective strategy for improving early detection and therefore decreasing healthcare system strain may also enhance policymaker engagement. Notably, policies must be tailored to account for heterogeneity across sub-Saharan Africa.

## Conclusion

Cancer stigma in sub-Saharan African countries will take significant and targeted work to overcome, as it is driven by complex cultural, social and structural factors that worsen mental health and lower survival rates. The heterogeneity of the region will require tailored, locally informed interventions. Stigma can directly impact a cancer patient’s access to employment or housing and significant work is urgently needed to develop, implement and sustain culturally informed, multilevel interventions grounded in local infrastructure and community networks. Health system capacity, cultural perceptions of mental illness and the availability of trained mental health workers will significantly determine how interventions can be feasibly delivered across different sub-Saharan African contexts, and any future interventions must account for these factors. Future interventions should be systematically evaluated across feasibility, effectiveness and implementation outcomes. Feasibility outcomes, such as patient and family acceptance of interventions and the capacity of health systems, should be analyzed. Effectiveness outcomes should measure whether interventions increase patient engagement and timely diagnoses, and implementation outcomes should measure whether interventions using the suggested multilevel framework can be sustained in a meaningful way.

As this is a perspective and does not employ a systematic search strategy, it may not describe all relevant possible avenues for interventions in sub-Saharan Africa. Furthermore, relatively few intervention studies exist in the region, limiting generalizability and requiring cautious interpretation. However, there is considerable opportunity for community-integrated healthcare given the structure of sub-Saharan African society and its collectivist nature. These efforts must be buttressed by sustained policy commitments and funding. Without tailored interventions to address the issue, cancer stigma will remain a key driver of poor mental health and decreased survival rates among cancer patients in sub-Saharan Africa.

## Data Availability

Data sharing is not applicable to this article as no datasets were generated or analyzed during the current study.

## Long descriptions

### Figure 1. Long description

The flowchart consists of three levels connected by arrows.

* Top Level: A yellow box labeled Societal and cultural factors. Three arrows point downward from this box to the middle row.

* Middle Level: Three boxes arranged horizontally.

1. Left (blue box): Enacted stigma (Mistreatment and discrimination from others).

2. Center (purple box): Anticipated stigma (Fear of stigma that may occur if one’s diagnosis is widely known).

3. Right (green box): Internalized stigma (Feelings of shame and self-blame).

* Bottom Level: A red box labeled Late presentation, screening avoidance, worse outcomes. Three arrows point from each of the middle boxes down toward this final box.

Navigate back to Figure 1..

### Figure 2. Long description

The diagram consists of four nested circles.

* At the center is a green circle labeled Individual. A line connects it to a box on the right containing: Mental health support and Peer support groups.

* The next layer outward is a yellow circle labeled Family. A line connects it to a box on the left containing: Caretaker support and Educational forums.

* The third layer is an orange circle labeled Community. A line connects it to a box on the right containing: Education led by C H Ws and C H Ws partners with leaders.

* The outermost layer is a purple circle labeled Policy. A line connects it to a box on the left containing: Intervention funding and Anti-discrimination policies.

Navigate back to Figure 2..
