# Supplementary material for: The growing and urgent need for interventions to mitigate cancer-related stigma in sub-Saharan Africa
Source: Glob Ment Health (Camb). 2026 Jul 20;13:e166. doi: 10.1017/gmh.2026.10252 (PMC13439423; doi:10.1017/gmh.2026.10252)
Supplement: Peer review history

# Reviewer report: Review: The growing and urgent need for interventions to mitigate cancer-related stigma in sub-Saharan Africa — R0/PR2

**DOI:** 10.1017/gmh.2026.10252.pr2

This is a well written review that makes a compelling case for interventions to mitigate cancer related stigma in Sub -Saharan Africa.

1. From the evidence adduced and the arguments advanced, it is apparent that policy makers have a crucial, if not the most crucial role in enabling the interventions suggested from funding to promulgating policies that help create a practice environment which enables cancer related stigma to be effectively addressed. It would therefore make the review even better if you add suggestions on how policy makers can be brought on board because without their buy in the status quo will remain even with well written papers like this.

2. The sentence (line 85-88) “In the context of sub-Saharan Africa, stigma around cancer is often driven by deeply rooted cultural misconceptions about the illness—for example, there are prevalent beliefs that religious punishment manifests as cancer, or that the disease can be contagious, spread through food or clothing” needs a reference.

# Reviewer report: Review: The growing and urgent need for interventions to mitigate cancer-related stigma in sub-Saharan Africa — R0/PR3

**DOI:** 10.1017/gmh.2026.10252.pr3

**Authors:** Donald Kokonya

**Affiliations:** School of Medicine, Masinde Muliro University of Science and Technology, Kenya

Comments on: The growing and urgent need for interventions to mitigate cancer-related stigma in sub-Saharan Africa

GMH-2025-0405

1. Missing citations need to be addressed

2. Long sentences need to be shortened and or split into two or more and be cited separately

3. Structural reorganization of the paper requires significant attention

4. Some literature is presented as if it is conclusion, instead being references

5. The write up is not presented conventionally in the required format -such no methods, no findings, no discussion

6. The paper requires significant attention

# Reviewer report: Review: The growing and urgent need for interventions to mitigate cancer-related stigma in sub-Saharan Africa — R0/PR4

**DOI:** 10.1017/gmh.2026.10252.pr4

The manuscript addresses an important and underexplored topic in global mental health; however, the central research question and its contribution relative to existing literature should be articulated more explicitly in the Introduction.

The rationale for the chosen study design and analytical framework requires clearer justification, particularly in relation to alternative approaches commonly used in this field.

Key concepts and constructs should be more precisely defined early in the manuscript to avoid ambiguity in interpretation, especially for readers from diverse disciplinary backgrounds.

The description of data sources would benefit from greater transparency regarding coverage, representativeness, and known limitations, particularly in low-resource settings.

Additional detail is needed on inclusion and exclusion criteria to clarify how the analytic sample was derived and to support reproducibility.

The methods section should more clearly explain how potential sources of bias (e.g., measurement error, missing data, or reporting heterogeneity across regions) were addressed or mitigated.

Statistical methods require more detailed reporting, including assumptions underlying the models used and how these assumptions were assessed.

The handling and interpretation of uncertainty should be strengthened, with clearer explanation of confidence or uncertainty intervals and their implications for inference.

Results would benefit from improved structure, with clearer separation between descriptive findings and model-based estimates.

The manuscript should more consistently distinguish between absolute measures and relative or standardized metrics to prevent misinterpretation.

Several key findings are presented with limited contextualization; stronger linkage to prior empirical evidence would enhance interpretability.

The Discussion occasionally overstates causal implications; language should be more carefully framed to reflect the observational nature of the data.

Greater attention should be given to heterogeneity across regions, populations, or subgroups, including clearer discussion of possible drivers of observed differences.

Limitations are acknowledged but could be expanded to more fully address issues of data sparsity, construct validity, and generalizability.

The policy and practice implications would benefit from sharper focus, prioritizing a smaller number of actionable recommendations grounded directly in the study’s findings.

Figures and tables are generally informative, but captions should be expanded to allow them to be interpreted independently of the main text.

Some figures appear dense and may benefit from simplification or redistribution to supplementary materials.

Terminology and notation should be standardized throughout the manuscript to improve clarity and coherence.

Redundancies between the Results and Discussion sections should be reduced to enhance readability.

A careful editorial review is recommended to address minor inconsistencies in formatting, references, and statistical reporting.

# Editor report: Recommendation: The growing and urgent need for interventions to mitigate cancer-related stigma in sub-Saharan Africa — R0/PR5

**DOI:** 10.1017/gmh.2026.10252.pr5

**Affiliations:** Psychiatry, Africa Institute of Mental and Brain Health, Kenya

The manuscript has an important topic with clear relevance to global mental health, policy, and cancer care in low resource settings. All three reviewers recognize the significance of the issue and the potential contribution of the review, particularly its emphasis on multilevel, culturally grounded interventions.

However, the reviewers raised some concerns that of the need for a major revision. Addressing these comments will enhance the manuscript clarity, credibility, and policy relevance.

Additionnaly to this:

-although the manuscript is labeled as a “Review”….., it does not describe how the literature was identified, selected, leaving readers unable to judge rigor and completeness. This is a substantive methodological weakness that prevents the paper from meeting expected standards for narrative, scoping or systematic reviews.

-In the first sentence “ Globally, stigma plays a significant yet largely unaddressed role in cancer mortality..” yet manuscript focuses almost exclusively on sub-Saharan Africa without contextualizing findings against global research trends.

-it does not provide actionable or evidence-based pathways forward. There is no categories of interventions are defined. This can be policy…… psychosocial.., clinical... No actors delivery platforms are identified example primary care, CHWs or the hospitals..

So I recommend Major Revision, and invite the authors to carefully and comprehensively revise the manuscript.

# Editor report: Decision: The growing and urgent need for interventions to mitigate cancer-related stigma in sub-Saharan Africa — R0/PR6

**DOI:** 10.1017/gmh.2026.10252.pr6

**Affiliations:** London School of Hygiene & Tropical Medicine, United Kingdom of Great Britain and Northern Ireland

No accompanying comment.

# Reviewer report: Review: The growing and urgent need for interventions to mitigate cancer-related stigma in sub-Saharan Africa — R1/PR8

**DOI:** 10.1017/gmh.2026.10252.pr8

Thank you for the revised manuscript.My comments have been adequatly addressed

# Reviewer report: Review: The growing and urgent need for interventions to mitigate cancer-related stigma in sub-Saharan Africa — R1/PR9

**DOI:** 10.1017/gmh.2026.10252.pr9

The Introduction should more explicitly situate the research question within current global mental health policy frameworks, clarifying how the findings may inform scaling of mental health services.

Greater clarity is needed regarding the theoretical or programmatic model underpinning the intervention or service being evaluated, including how it aligns with existing mental health care frameworks.

The description of participant recruitment procedures should be expanded to clarify whether consecutive, convenience, or purposive sampling strategies were used.

The manuscript should more clearly state how participant retention was monitored and whether attrition may have influenced the final analytic sample.

The authors should provide additional detail regarding fidelity of implementation, including whether standardized procedures or monitoring mechanisms were used to ensure consistent intervention delivery.

Greater attention should be given to contextual factors (e.g., health system capacity, cultural perceptions of mental illness, workforce availability) that may influence implementation outcomes.

The manuscript would benefit from clearer differentiation between feasibility outcomes, effectiveness outcomes, and implementation outcomes.

The measurement tools used to assess mental health outcomes should be more thoroughly described, including information on cultural adaptation, translation procedures, and psychometric validation.

The statistical analysis section should clarify whether clustering or site-level variation was considered, particularly if data were collected across multiple facilities or communities.

The manuscript would be strengthened by including a brief justification for the chosen follow-up duration and its adequacy for capturing meaningful mental health change.

The Results section should more clearly prioritize the primary outcomes, as currently several secondary findings receive comparable emphasis.

The authors should clarify whether any subgroup analyses were prespecified or conducted post hoc.

Greater transparency is needed regarding potential sources of implementation variability across sites or providers.

The Discussion should more explicitly consider alternative explanations for the observed outcomes, including contextual changes unrelated to the intervention.

The manuscript would benefit from a deeper discussion of scalability and sustainability, particularly with respect to financial, workforce, and health system constraints.

Equity considerations should be more explicitly addressed, including whether the intervention reached underserved or marginalized populations.

The limitations section should discuss the potential influence of participant expectations or provider enthusiasm on reported outcomes.

The policy implications should be more cautiously framed, emphasizing the need for replication in diverse contexts before large-scale adoption.

The conclusions would be strengthened by more clearly distinguishing between findings supported directly by the data and broader programmatic recommendations.

Tables and figures could be refined to improve readability, particularly by simplifying dense data presentations.

The manuscript would benefit from a brief statement regarding data transparency and availability to enhance reproducibility.

# Editor report: Recommendation: The growing and urgent need for interventions to mitigate cancer-related stigma in sub-Saharan Africa — R1/PR10

**DOI:** 10.1017/gmh.2026.10252.pr10

**Affiliations:** Psychiatry, Africa Institute of Mental and Brain Health, Kenya

The manuscript has improved and addresses several of my comments, particularly regarding intervention frameworks and policy relevance. However, the absence of any description of how literature was identified remains a significant methodological gap, even for a narrative review. Most narrative reviews include a brief description of how literature was identified, such as: databases searched, search terms, time frame, inclusion focus (e.g., stigma, oncology, mental health)

# Editor report: Decision: The growing and urgent need for interventions to mitigate cancer-related stigma in sub-Saharan Africa — R1/PR11

**DOI:** 10.1017/gmh.2026.10252.pr11

**Affiliations:** London School of Hygiene & Tropical Medicine, United Kingdom of Great Britain and Northern Ireland

No accompanying comment.

# Editor report: Recommendation: The growing and urgent need for interventions to mitigate cancer-related stigma in sub-Saharan Africa — R2/PR13

**DOI:** 10.1017/gmh.2026.10252.pr13

**Affiliations:** Psychiatry, Africa Institute of Mental and Brain Health, Kenya

The author has addressed all the reviewer comments adequately and substantially improved the manuscript. Only minor revisions are needed, such as tightening the language for clarity and formatting inconsistencies.

# Editor report: Decision: The growing and urgent need for interventions to mitigate cancer-related stigma in sub-Saharan Africa — R2/PR14

**DOI:** 10.1017/gmh.2026.10252.pr14

**Affiliations:** London School of Hygiene & Tropical Medicine, United Kingdom of Great Britain and Northern Ireland

No accompanying comment.

# Editor report: Recommendation: The growing and urgent need for interventions to mitigate cancer-related stigma in sub-Saharan Africa — R3/PR16

**DOI:** 10.1017/gmh.2026.10252.pr16

**Affiliations:** Psychiatry, Africa Institute of Mental and Brain Health, Kenya

I have carefully reviewed the revised manuscript “The growing and urgent need for interventions to mitigate cancer-related stigma in sub-Saharan Africa” and I am satisfied with the current version of the manuscript. The authors have adequately addressed the concerns raised during the review process, and the manuscript is now clear, coherent, and of relevance to the field.

In my opinion, the manuscript makes a meaningful contribution to the literature on cancer-related stigma and public health in sub-Saharan Africa. I therefore recommend the manuscript for publication in its current form.

# Editor report: Decision: The growing and urgent need for interventions to mitigate cancer-related stigma in sub-Saharan Africa — R3/PR17

**DOI:** 10.1017/gmh.2026.10252.pr17

**Affiliations:** London School of Hygiene & Tropical Medicine, United Kingdom of Great Britain and Northern Ireland

No accompanying comment.
